# Active Solid-State Nanopores: Self-Driven Flows/Chaos at the Liquid–Gas Nanofluidic Interface

**DOI:** 10.1021/acs.langmuir.3c02776

**Published:** 2023-11-29

**Authors:** Vinitha Johny, Siddharth Ghosh

**Affiliations:** † International Center for Nanodevices, INCeNSE-TBI, Indian Institute of Science Campus, Bangalore 560 012, Karnataka, India; ‡ Open Academic Research Council, Hooghly 712 235, West Bengal, India; § Open Academic Research UK CIC, Cambridge CB3 1AT, U.K.; ∥ International Center for Nanodevices, High Tech Campus Eindhoven, Eindhoven 5656 AE, The Netherlands; ⊥ Department of Applied Mathematics and Theoretical Physics, University of Cambridge, Cambridge CB3 0WA, U.K.

## Abstract

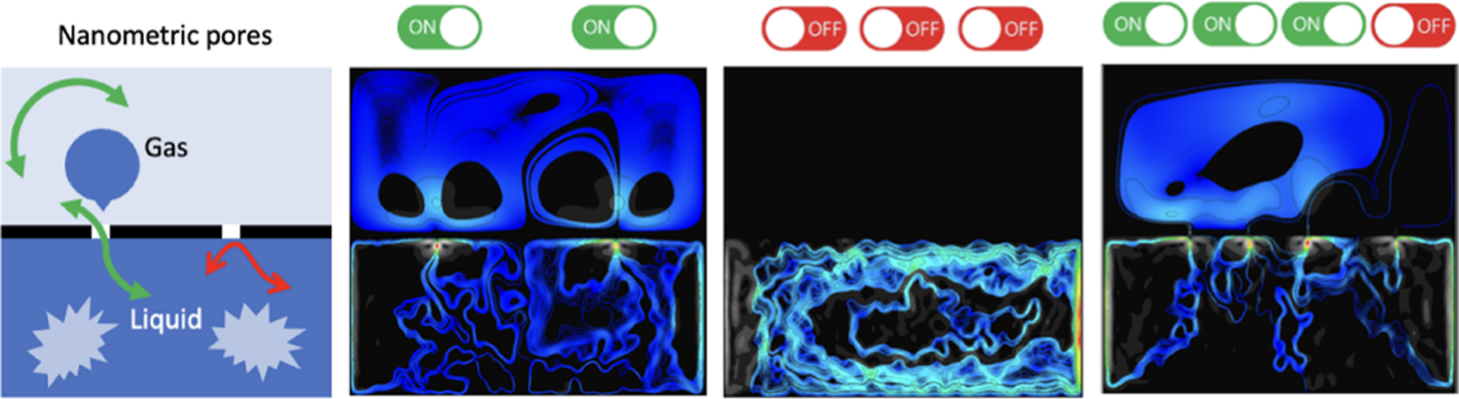

Here, we present a comprehensive study of self-driven flow dynamics at the liquid–gas interface within nanofluidic pores in the absence of external driving forces. The investigation focuses on the Rayleigh–Taylor instability phenomena that occur in sub-100 nm scale fluidic pores interfacing between 2 μm scale water and air reservoir. We obtain a flow velocity equation, and we validate it using simulations, concentrating on the mass transfer efficiency of these flow structures. Furthermore, we introduce the concept—“active solid-state nanopore”—that exhibits a self-driven flow switching behavior, transitioning between active and passive states without the need for mechanical components. We found a unique state of chaos at the nanoscale resembling the chaotic motion of fluid. This study contributes to the preliminary understanding of fluid dynamics at the classical–quantum interface. Implications of self-driven nanofluidics extend across diverse fields from biosensing and healthcare applications to advancing net-zero sustainable energy production and contributing to the fundamental understanding of fluid dynamics in confined spaces.

## Introduction

Nanofluidic pores,^1−4^ which refer to sub-100 nm scale conduits for fluid flow, have attracted significant attention for electrical energy harvesting.^5,6^ However, the exploration of mechanical energy extraction from nanofluidics remains a relatively uncharted territory. Intriguingly, there is evidence that nanofluidic systems can generate substantial forces, as exemplified by the flexible nanopores found in the stomata of leaves. These natural nanopores create remarkable negative pressure, on the order of megapascals, enabling them to transport water against gravity over substantial heights.^4,7^ In this study, we delve into the concept of mechanical energy extraction from nanofluidics, specifically focusing on a transpirational approach.^8,9^ This approach, inspired by nature, holds the promise of scalable designs suitable for very large scale integration/VLSI applications,^10^ enabling on-chip flow generation,^11^ molecule sampling,^12^ and nanorobotics.^13^ Furthermore, it aligns with the pursuit of clean energy solutions that offer precise fluid control at the scale of biological cellular nanopores, propelling us closer to the realm of artificial life and next-generation nanomechanically engineered transportation.^14−16^ To advance our understanding of interfacial fluid dynamics within nanometric fluidic pores, we turn to the phenomenon of Rayleigh–Taylor instabilities. These instabilities emerge when two fluids of differing densities intersect,^17^ giving rise to intricate interference patterns at their interface due to molecular interactions.^18^ By examining the rate at which these instabilities develop, we gain valuable insights into the fluid dynamics of interfacial systems.^19^ Remarkably, fluid dynamics in nanofluidic pores at the liquid–gas interface have remained relatively unexplored. This gap in research is not solely due to experimental challenges but also arises from theoretical complexities.

**Figure 1:**
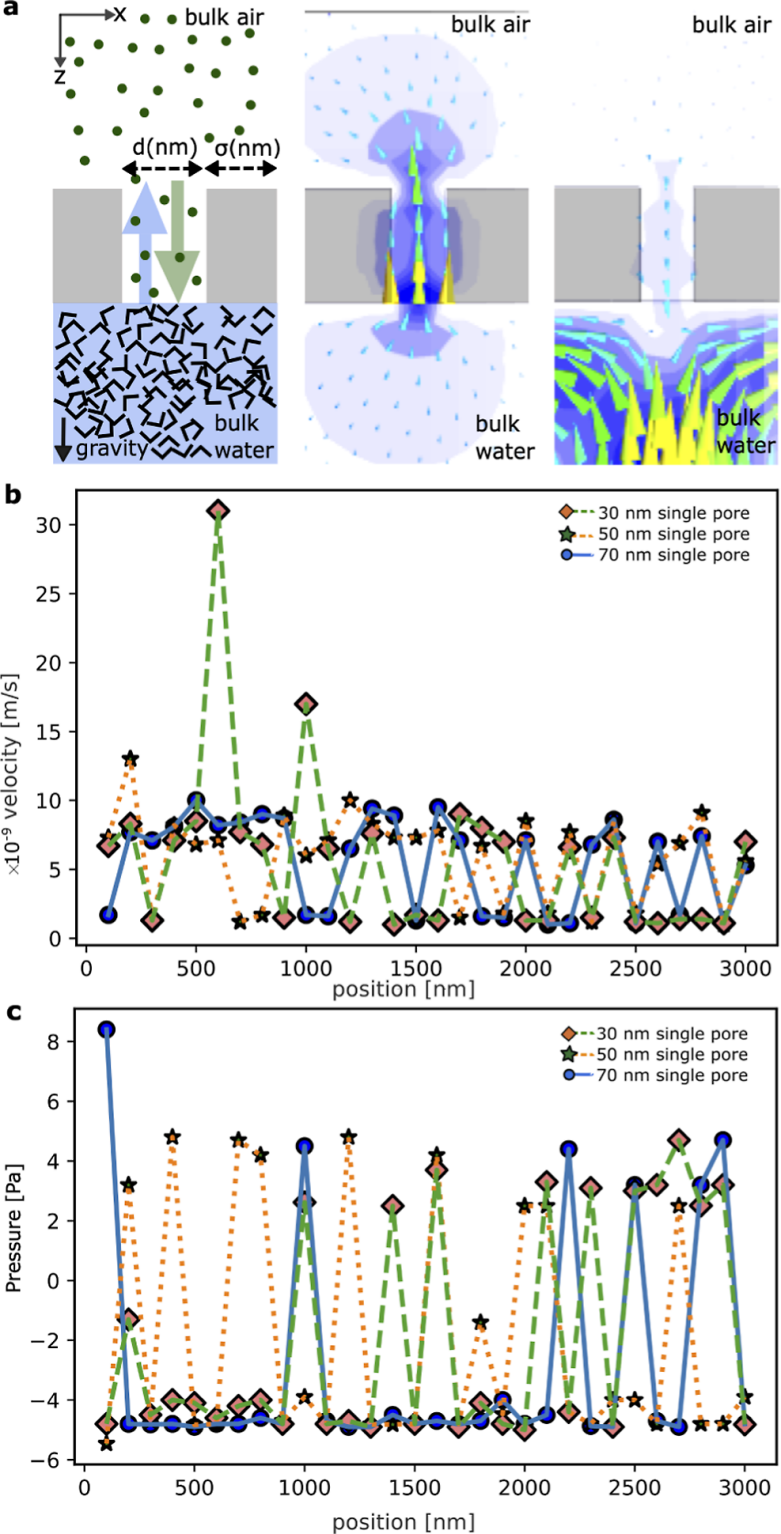
(a) Principle of self-driven flow at the interface of air and water through a single nanofluidic pore; σ (nm) is the distance of nanofluidic pore from the boundary, *d* (nm) is the nanofluidic pore throat diameter. (b) Chaotic oscillation of reference maximum velocity in the mixed phase of water and air at the interface with the single pore of *d* of 30, 50, and 70 nm. (c) Variation of pressure inside the nanofluid due to the distance from edge, σ.

Herein, we present a novel study of self-driven flow dynamics through sub-100 nm nanofluidic pores, drawing inspiration from the behavior of nanopores on leaf surfaces. Our investigation employs a Rayleigh–Taylor instability model at the water–air interface to elucidate the nontrivial phenomenon of evaporation against gravity through nanofluidic pores, mirroring the mechanisms at play in natural leaf surfaces. Notably, we unveil the dynamics of flow in stable solid-state nanofluidic pores without the application of an external voltage or pressure against gravity. While conventional nanofluidic transport mechanisms have primarily revolved around electrokinetic and pressure-driven methods,^20,21^ the transpirational approach, as observed in Figure 1a, introduces unique factors such as van der Waals forces, electrostatic interactions, induced dipoles between polar and nonpolar species, and oscillatory solvation pressure. The results of our study reveal both linear and chaotic flow patterns in microreservoirs featuring nanofluidic pores with significant variations in velocity across different pore positions. The findings of this research not only uncover the exotic fluid dynamics governing sub-100 nm nanofluidic pores but also open up exciting possibilities for engineering self-driven nanopores using solid-state materials. This pioneering exploration prompts new questions about interfacial hydraulic resistance, which may find relevance in understanding phenomena such as stomatal closure in plants, and sparks innovative ideas for nanoengineered propulsion in transportation.^22^

## Experimental Section

The model investigates fluid behavior at the nanofluidic liquid–gas interface between bulk water and bulk air. Figure 1a provides an overview of the system with unidirectional flow patterns. To set the stage, we consider a fluidic reservoir measuring 6000 × 6000 nm^2^, divided by a 100 nm wall. This silicon wall comprises 96% of the separation at the water–air interface. Key material properties include a density of 2.18 × 10^–12^ g/m^3^, elastic modulus of 6.8 × 10^10^ Pa, shear modulus of 2.8 × 10^10^ Pa, Poisson’s ratio of 0.19, thermal conductivity of 1.38 W/m·K, and specific heat of 750 J/kg·K. We utilize a triangular mesh geometry with an average element area of 9 × 10^–12^ m^2^ and an element mesh size of 2.5 × 10^–8^ m. The viscous model is set to laminar, and an implicit formulation is employed. The system temperature is maintained at 288.16 K. Our model is based on the Navier–Stokes equations, which consist of the continuity, momentum, and energy equations. Additionally, we employ a volume fraction equation to account for the multiphase flow. The general fluid transport equation is given as
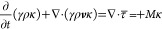
1Here, γ represents the phase volume fraction, ρ is the phase density, κ is the phase variable, ***v*** is the phase velocity, *M* is the source term for added mass transfer, and τ̿ is the diffusion term (stress tensor). The volume fraction equation is described as
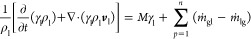
2This equation considers mass transfer through the nanofluidic pores regardless of the flow direction. To study the system’s behavior, we vary the diameter of single nanofluidic pores (*d*) at 30, 50, and 70 nm. We also vary the distance (σ) from the boundary of the reservoir in the orthogonal direction to gravity in 100 nm steps from 100 to 3000 nm at the center. It is important to note that the mesoscopic physics of the fluidic interface at the nanometric length scale adds complexity to our study. All simulations were performed in two-dimensional and performed using finite element method Ansys Fluent 2021 R1 solver in Intel i7 CPU.

## Results and Discussion

### Single Nanofluidic Pore at Liquid–Gas Interface

**Figure 2:**
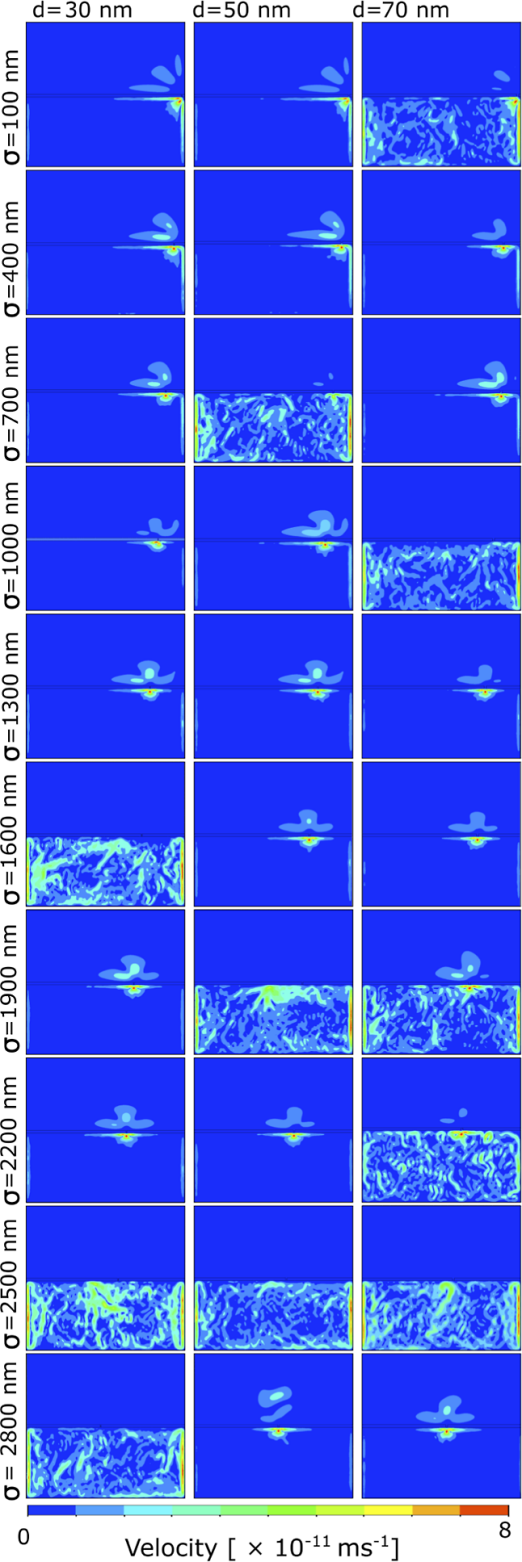
Pattern of flow dynamics in bulk water and bulk air due to single nanopores by varying its diameter, *d* and position from the boundary, σ—column 1, column 2, and column 3 are for diameters 30, 50, and 70 nm, respectively. In all cases, σ is varied from the 100 to 2800 nm referring position from the boundary to the center of the systems.

Although the physical model of the leaf-like system shown in Figure 1a appears geometrically simple, the fluid mechanics revealed nonintuitive behavior that defies a linear trend. One might expect the maximum velocity of fluid flow will occur at the nanofluidic interface, resulting in a plateaued velocity profile. However, as shown in Figure 1b,c, we observe strong fluctuations in reference velocities and pressure. For a 30 nm nanofluidic pore, varying the pore’s position by 200 nm produces velocity steps of 0, 4.5 × 10^–9^, and 30 × 10^–9^ m/s. Similarly, in a 50 nm nanofluidic pore system, we observe velocity steps of 0 and 10 × 10^–9^ m/s within a 100 nm distance. Surprisingly, there is no flow between 2500 and 2900 nm, followed by a sudden return of flow at 3000 nm. This strong oscillation nearly stabilizes within the range of 900 to 1600 nm, with velocities ranging from 4.5 × 10^–9^ to 10 × 10^–9^ m/s, only to continue with strong oscillations over distance. A similar pattern is observed in the 70 nm nanopore system within σ = 100 nm. A nearly stable plateau with an average velocity of 7.5 × 10^–9^ m/s can be observed within σ = 200 to 900 nm, followed by continued strong fluctuations. The aforementioned oscillations prompted us to investigate the overall flow patterns of the system. Figure 2 illustrates the distribution of velocity magnitude over two spatial dimensions, *x* and *y*. The most common flow pattern in single nanofluidic pore systems resembles a cloud or hat shape with large flow velocities. The movement of liquid from the water phase to the air phase encounters a hindrance by back-flow, as depicted in Figure 1c, often resulting in the formation of a visible meniscus. Interestingly, we observe the sudden collapse of flow in the air region at certain positions of nanofluidic pores, varying the distance from 100 to 3000 nm. A driving mechanism that initiates and inhibits flow appears to be continuous. The pressure drops above the meniscus induce back-flow in the water phase, initiating motion. This phenomenon is observed in several instances across different pore sizes and positions. In Figure 2, we notice distinct small peaks of counter-flow on either side of the highest peak, similar to the Gibbs–Marangoni flow.^23^ The maximum flow velocity through 30, 50, and 70 nm nanofluidic pores are found to be approximately 1.3 × 10^–11^, 1.3 × 10^–9^, and 8.4 × 10^–9^ m/s, respectively, when the reference flow velocity through the nanofluidic pore is set to be 4.2 × 10^–9^ m/s.

#### Zero-Flow Nanochaos

In several cases, we observed no flow in the air phase, while the water phase exhibited significant chaotic motions with strong velocity components near the boundary. We have termed this phenomenon “zero-flow nano-chaos” (0FNC state). For instance, in Figure 2, the 0FNC state is evident in the 30 nm nanofluidic pore when positioned at 1600, 2500, and 2800 nm from the right boundary. Figures S1 and S2 further illustrate discrete events of the 0FNC state at various positions, such as 300, 1200, 2000, and 2300 nm and a range from 1400 to 1600 nm, as well as from 2500 to 2900 nm. While 50 and 70 nm nanofluidic pores do not consistently exhibit the 0FNC state at the same positions as the 30 nm pore, they do display this behavior at specific locations. Figure S1 demonstrates events of the 0FNC state for the 50 nm nanofluidic pore at 700, 1900, and 2500 nm. At σ = 700 nm, we noted a transition with a small flow component in the air phase. At σ = 2500 nm, the 0FNC state was observed in all cases. The direction of vortices and their pairs may have a certain relation with the 0FNC state, a topic we discuss in the subsequent Results and Discussion section. Figure S1 reveals a range of positions within σ = 2500–2900 nm where the 50 nm nanofluidic pore experiences three events of flow in the air phase, breaking the 0FNC state. Similarly, Figure S2 demonstrates a pattern similar to that of the 30 nm pore for the 70 nm pore, except at σ = 2600 nm, where we found a transition from the 0FNC state to flow in the air.

### Multiple Nanofluidic Pores at Liquid–Gas Interface

**Figure 3:**
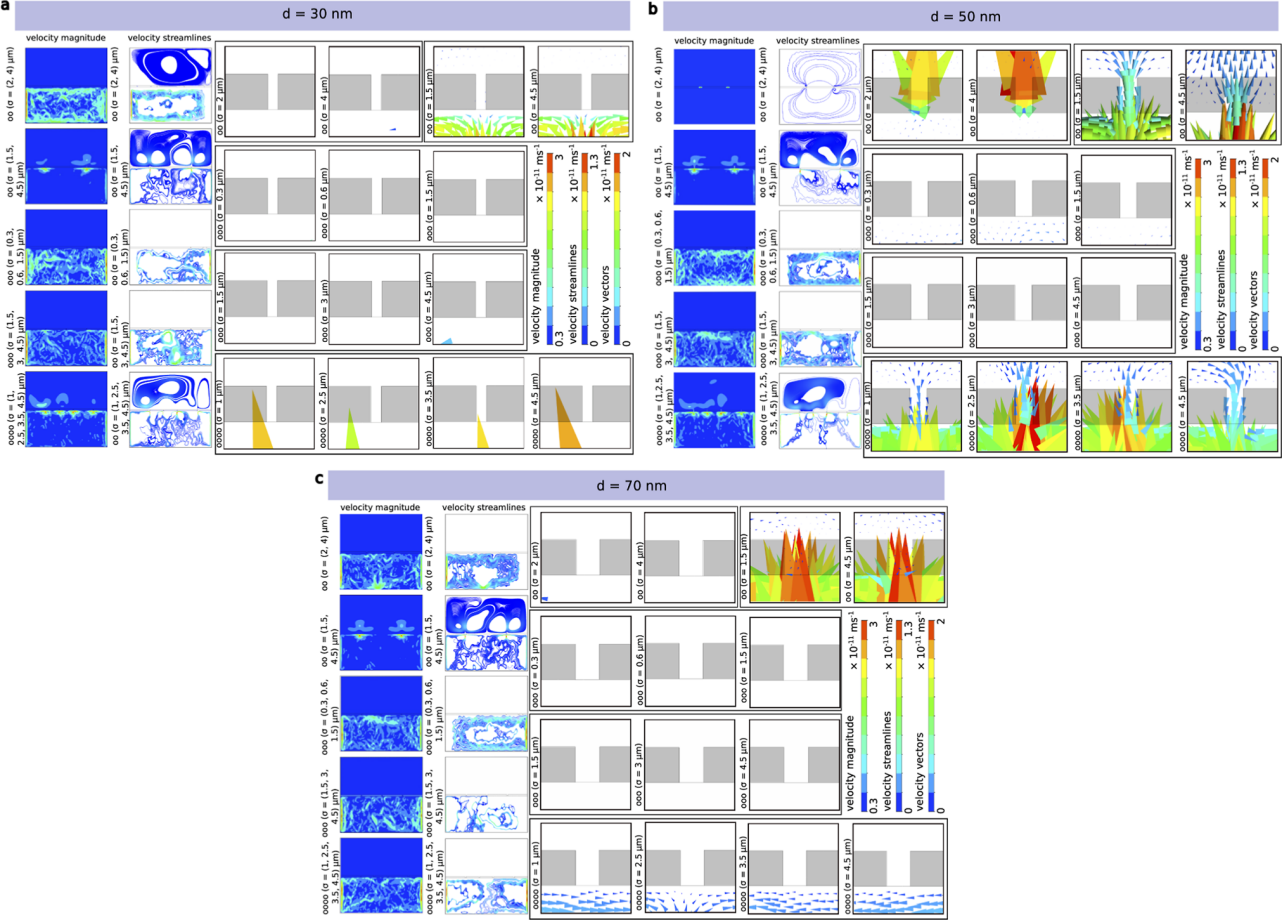
Flow patterns of multiple nanofluidic pore systems. Global/complete views of velocity flow pattern with velocity vectors in the first column and its corresponding velocity stream lines in the second column, and followed by these global views, fourth to seventh columns represent local components of velocity vectors of local flow at the individual nanofluidic pores in the same order of their presence from left to right in the global view. (a) 30 nm showing no prominent flow from one fluid phase to another in two pores (oo), three pores (ooo), and four pores (oooo), (b) 50 nm with two pores (oo) possessing strong flow from water to air as well as moderate flow from air to water, three pores (ooo) with no flow from different fluid phases, and four pores (oooo) showing weak flow components from one fluid phase to another, and (c) 70 nm with no flow component from one fluid phase to another in two pores (oo), three pores (ooo), and four pores (oooo).

We have conducted an investigation into the behavior of multiple nanofluidic pore systems at the liquid–gas interface. In Figure 3, we present the flow behavior of these systems, varying the number of nanofluidic pores from two to four, while studying their spatial dependencies. Two cases of two-pore systems are examined based on pore positions: σ = (2, 4) μm and σ = (1.5, 4.5) μm. For three-pore systems, variations include σ = (0.3, 0.6, 1.5) μm and σ = (1.5, 3, 4.5) μm. In the case of four-pore systems, we focused on one scenario with σ = (1, 2.5, 3.5, and 4.5) μm. We provide global views of velocity flow patterns with velocity vectors in the first column and corresponding velocity streamlines in the second column for three nanofluidic pore sizes: 30 nm (Figure 3a), 50 nm (Figure 3b), and 70 nm (Figure 3c). Subsequently, we present local components of the velocity vectors for each nanofluidic pore. In two-pore systems, both flow and 0FNC state are observed (Figure 3a). For the three-pore systems, 0FNC conditions persist. In the streamline visualization, we observe clustered flow in the central region of the water phase. Magnified views of these pores do not show flow components inside the pore. For four-pore systems, flow is visible through all pores with clustered flow streamlines oriented toward the air phase from water to air phases.

#### 30 nm Multipore Systems

In two-pore systems (Figure 3a.oo), we observe both the 0FNC state and fully developed flow behavior. Notably, when the pore spacing (σ) is set to (2, 4) μm, 0FNC predominates within the water phase. A closer inspection of pores at σ = 2 μm and σ = 4 μm (Figure 3a.oo) reveals the absence of flow at the water–air interface within the pores. However, by adjusting the pore positions closer to the nearest boundaries (by 500 nm) at σ = (1.5, 4.5) μm, the system transitions into a state of flow, with flow rates of 2.4 × 10^–11^ and 3 × 10^–11^ m/s through the first and second pores, respectively. The velocity streamlines indicate a synchronized global flow between the two pores. In the magnified views of pores at σ = 1.5 μm and σ = 4.5 μm, minor flow components are observed at the water–air interface within the pores, with significantly larger flow components near the pores in the water phase. Expanding to three-pore systems (Figure 3a.ooo), we again observe the 0FNC condition. Streamline visualizations at σ = (1.5, 3.0, and 4.5) μm show clustered flow in the central region of the water phase. However, magnified views of groups of three sets of pores in both cases do not display any flow components within the pores (e.g., Figure 3a.ooo at σ = (0.3, 0.6, and 1.5) μm and Figure 3a.ooo at σ = (1.5, 3, and 4.5) μm). Further increasing the number of pores to four (Figure 3a.oooo), flow is visible through all pores, with clustered flow streamlines oriented toward the pores and the air phase, transitioning from the water phase to the air phase. In the magnified view of these pores at σ = (1, 2.5, 3.5, and 4.5) μm, velocity vectors within each pore exhibit orientation toward the air phase (forward flow). Notably, the number of pores displaying forward flow vectors exceeds those with backward flow vectors (toward the water phase), explaining the higher total flow rate toward the air phase.

#### 50 nm Multipore Systems

When we increased the pore size to 50 nm, the 0FNC state decreased. Flow components toward the air phase are observed when σ = (2 and 4) μm. Changing the position of two pores toward the nearest boundaries (σ = (1.5 and 4) μm) results in reversed flow toward the water phase through one pore and a 2π transition toward the air phase through the other. For three-pore systems, the 0FNC state is prominent in the water phase. Magnified views of these pores do not show flow vectors inside the pores. However, in four-pore systems, a continuous and prominent flow through all pores is observed.

As shown in Figure 3b, we investigate all the same positions of 30 nm systems with a change in pore size to 50 nm. The 0FNC systems have decreased as the pore size increased from 30 to 50 nm. When σ = (2 and 4) μm, the flow components are significant toward the air phase. The magnified view of these pores as in Figure 3b.oo (σ = 2 μm) and Figure 3b.oo (σ = 4 μm) shows a high magnitude stream of velocity vectors toward the air phase. As the position of each nanopore is changed by 500 nm toward their nearest boundaries, the flow dynamics is observed to change considerably as in Figure 3b.oo (σ = (1.5 and 4) μm). In the magnified view of the pore in Figure 3b.oo (σ = 1.5 μm), the flow vectors are oriented toward the water phase. Here, a reverse flow toward the water phase with magnitude of 1.5 × 10^–11^ m/s is observed. In Figure 3b.oo (σ = 4 μm), the flow vectors take a 2π transition toward the air phase. When the number of pores is increased to three, 0FNC is prominent in the water phase, as shown in Figure 3b.ooo. The magnified pore views of three-pore systems of pore size 50 nm in Figure 3b.ooo (σ = (0.3, 0.6, and 1.5) μm) and Figure 3b.ooo (σ = (1.5, 3, and 4.5) μm) do not show any flow vectors inside the pore. Figure 3b.oooo shows evident velocity vector components through the initial three pores from the left boundary which tend to cease at the fourth pore. In the magnified view of these pores, Figure 3b.oooo (σ = 1 μm) and (σ = 4.5 μm) have a flow magnitude of 1 × 10^–11^ m/s toward the water phase or reverse flow, and Figure 3b.oooo (σ = 2.5 μm) and (σ = 3.5 μm) have a flow with greater magnitudes of 3 × 10^–11^ and 2.7 × 10^–11^ m/s toward the air phase.

#### 70 nm Multipore Systems

In systems with 70 nm pores, the 0FNC state is prominent in the water phase when σ = (2, 4) μm. Changing the position of two pores toward the nearest boundaries results in maximum velocity components toward the air phase. A magnified view shows minor flow components inside the pores toward the water phase. For three-pore systems, there are no flow vectors inside the pores, irrespective of pore position. In four-pore systems, there are no flow components through any of the pores.

The decrease in 0FNC, when the pore size increased from 30 to 50 nm, is collapsed when the pore size is further increased to 70 nm in Figure 3c. When σ = (2 and 4) μm, 0FNC is prominent in the water phase. A magnified view of the pores is shown in Figure 3c. When σ = 2 μm, Figure 3c. When σ = 4 μm, no flow is shown at the interface of the water–air in the pores. In Figure 3c.oo (σ = (1.5, 4) μm), the system shows maximum velocity components with magnitude of 3 × 10^–11^ m/s through the nanopore toward the air phase. A magnified view of these pores also shows minor flow components of magnitude 0.3 × 10^–11^ m/s inside the pores toward the water phase (reverse flow). As the number of pores increased to three, there are no flow vectors inside the pores. In the magnified view of the pores in Figure 3c.ooo (σ = (0.3, 0.6, 1.5) μm), there are minor flow vectors of magnitude 0.3 × 10^–11^ m/s in the water phase and, in Figure 3c.ooo (σ = (1.5, 3, 4.5) μm) no flow vectors in water phase is observed. When the number of pores is increased to four, there are no flow components through any of the pores in Figure 3c.oooo. In the magnified view of the pores as in Figure 3c.oooo (σ = 1.5 μm), the velocity vectors are oriented toward the right boundary, whereas in σ = 2.5 μm, the flow vectors diverge near the nanopore in the water phase. In Figure 3c.oooo (σ = (3.5, 4.5) μm), the flow vectors are oriented to the left boundary in the magnified view of the pore. The change in orientation of these flow vectors are due to the divergence of flow components as observed in Figure 3c.oooo (σ = (1, 2.5, 3.5, 4.5) μm) of velocity streamlines.

The observed behavior in multipore systems differs from single-pore systems, emphasizing the complexity of these systems. The influence of the pore position in switching between the flow and 0FNC state is evident. Further investigation is needed to fully understand the dynamics of multipore systems. In four-pore systems with specific pore distances, continuous flow is observed, regardless of pore size for 30 and 50 nm pores. However, when the pore size is increased to 70 and 100 nm, 0FNC states (Figures S4 and S6) are observed, contrary to single-pore systems. This challenges the generalization from single-pore systems and highlights the importance of pore arrangement. The complete flow dynamics of multipore systems remains complex and requires further investigation.

### Analytical Approach

Let us now analytically validate the transport mechanisms and flow structures, as shown in Figure 2. We used densities of two fluids as ρ_g_ = 1.225 kg/m^3^ for air and ρ_l_ = 997 kg/m^3^ for water. Since the domain is larger than the wavelength of the instabilities, the velocity potential of the motion is given by Taylor^19^ as
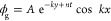
3
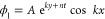
4where ϕ_g_ is the velocity potential in air, *A* is the amplitude, *k* is the wavenumber, *n* is an integer, and ϕ_l_ is the velocity potential in water. We assume that acceleration takes place toward the denser fluid. At the interface
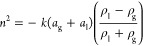
5

In eq 5, the terms *a*_g_ and *a*_l_ represent accelerations in the gaseous and liquid phases, respectively. These accelerations can be attributed to the effects of gravity, which are essential in understanding the behavior of the Rayleigh–Taylor instability. The inclusion of these terms is consistent with the classical fluid dynamics theory, which accounts for the influence of gravity on fluid flow phenomena. Moreover, it is important to note that the acceleration terms are critical in determining the growth and behavior of the instability. They represent the rate of change of the velocity in response to the density difference between the two phases. While this explanation does not delve into the specifics of how the accelerations are calculated, it establishes their theoretical foundation in the context of Rayleigh–Taylor instability, which is a well-established phenomenon in fluid dynamics. We acknowledge that in the context of our nanometric geometry, the application of established equations such as the Poiseuille equation and Taylor’s characteristic equation may raise questions. However, the Poiseuille equation is commonly applied to describe the flow in micro- and nanoscale channels under certain conditions, including laminar and fully developed flows.^24−27^ While our system features nanometric geometries, the use of the Poiseuille equation is justified by the laminar nature of the flow and the steady-state conditions achieved during our simulations. We acknowledge that these conditions are essential for the equation’s validity and emphasize that our system meets these criteria. Taylor’s characteristic equation, derived with the presence of an external acceleration superimposed on gravitational acceleration, is applied here to explain the dynamics in our nanoscale system. The acceleration terms in the equation account for the effects of gravity on the Rayleigh–Taylor instability, which is essential to our analysis. We acknowledge that the system lacks an external acceleration component but emphasize that the equation’s applicability is justified by its utility in elucidating the observed phenomena in our nanoscale setup.

In our study, we observe the phenomenon of the 0FNC state, which represents a unique behavior within nanofluidic pores. This term signifies that despite the absence of a conventional macroscopic flow, the system exhibits complex and seemingly chaotic fluid dynamics at the nanoscale. The use of established equations like the Poiseuille equation and Taylor’s characteristic equation may seem unconventional in the context of the 0FNC state. However, it is important to note that these equations offer valuable insights into the underlying physics of the system, even in situations where macroscopic flow appears to be absent. The 0FNC state is a manifestation of intricate molecular-scale interactions that are not readily captured by conventional continuum mechanics. While the conditions for applying these equations may seem unusual given the presence of the nanochaotic behavior, we emphasize that our objective is to gain a deeper understanding of the fundamental principles governing fluid dynamics at the nanoscale. The application of established equations serves as a bridge between the macroscopic and molecular realms, allowing us to uncover hidden complexities in nanofluidic systems. A single nanofluidic pore with diameter *d* = 30 nm and maximum flow velocity will have *n*^2^ = −1.2 × 10^–6^*k*. This notation was chosen for its convenience in representing the relationship among these variables in our context. While unconventional, it accurately reflects the key aspects of our analysis and allows for a more intuitive understanding of the system. Since (*a*_g_ + *a*_l_) is positive, the flow instability evolves over time, initially exhibiting transient behavior until it stabilizes into a consistent pattern. These behaviors can be described by a relationship proportional to , which is unity in our system and explains the no-flow systems. For systems without flow, eq 2 simplifies to
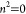
6

In the water phase, the displacement of instability is given by
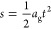
7where *t* is the time taken for the steady state to be reached from the initial time, *a*_g_ is the acceleration of the molecules in the gaseous phase, and ***v*** is the velocity of flow. In an evaporating liquid–gas interface, a certain number of molecules condense onto the surface of the liquid, which can result in backflow in some cases. The ratio of atoms condensing to atoms evaporating is unclear and can be complicated to predict from system to system. The number of condensing molecules is a function of the mean velocity through the nanofluidic pore. The number of evaporating particles can be described by Feynman^28^ as
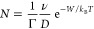
8where ν is the average speed of particles, *W* is the extra energy needed for evaporation, *k*_B_ is the Boltzmann constant, and Γ is the cross-sectional area. However, since we describe the system using continuum mechanics, we do not pursue this quantum mechanical perspective. The vapor transport through the nanofluidic pore is governed by the conventional Hagen–Poiseuille equation^29^
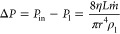
9where *P*_in_ is the pressure at the interface, *P*_l_ is the liquid phase pressure, *ṁ* is the mass flow rate through the nanofluidic pore, η is the viscosity of the liquid, *r* is the radius of the nanofluidic pore, *L* is the flow length, and ρ is the liquid density. Since we are addressing transport in nanofluidic pores using the Rayleigh–Taylor instability
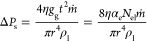
10where Δ*P*_s_ is the pressure drop in simulations, α_e_ is the element size of the geometry used for simulation, and *N*_e_ is the number of elements in the flow region. Then, the simulation velocity is given by
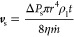
11

**Figure 4:**
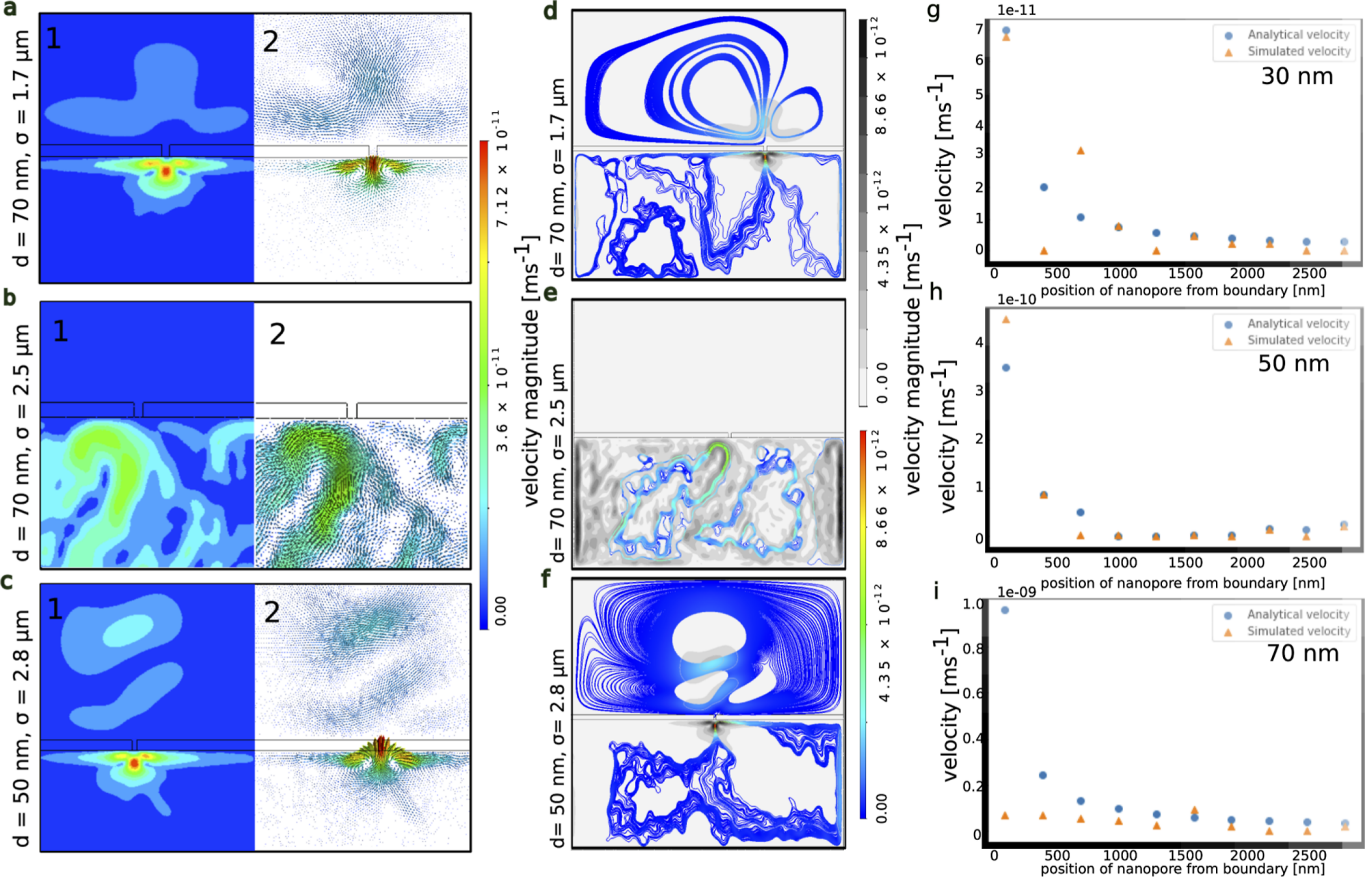
Exemplary local flow patterns at the vicinity of the nanofluidic pore. (a) Flow dynamics of a single nanofluidic pore system with *d* = 70 nm situated at σ = 1700 nm; (1) velocity magnitude distribution and (2) velocity vectors indicating vortices in water and air resulting with no flow from water to air. Have (b) single nanofluidic pore system with *d* = 70 nm situated at σ = 2500 nm; (1) velocity magnitude with high value near the opening and (2) velocity vector with no flow in air but flow component. (c) 50 nm single nanofluidic pore system at σ = 2800 nm; (1) velocity magnitude and (2) velocity vector with prominent back flow and vertices in water and air. Global flow patterns and velocity streamlines in the systems for the single nanopore. (d) Complete view of (a) showing no connection of streamlines from water to air and vice versa. (e) Complete view of (b) with flow in air, significant chaotic flow patterns in air phase, and strong velocity magnitude at the boundary. (f) Complete view of (c) with the largest velocity magnitude near nanofluidic pore in water and cavitation being formed in the air phase. Analytical and simulated mass flow velocity through nanopore as a function of σ. (g) *d* = 30 nm with prominent errors for σ at 400 and 700 nm, (h) *d* = 50 nm with prominent errors for σ at 100, 700, and 1300 nm, and (i) *d* = 70 nm with prominent errors in all σ except 1600 nm.

The analytical form of Δ*P* is given by
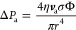
12and the corresponding velocity is given by
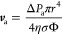
13where σ is the distance of the nanopore from the boundary of the system, Φ is the area of the nanopore, and Δ*P*_a_ is the pressure drop across the interface, which is a constant. In Figure 4g–i, we observe certain agreements between analytical and numerical velocities, except in a few cases, where large errors are observed.

### Flow Physics

**Figure 5:**
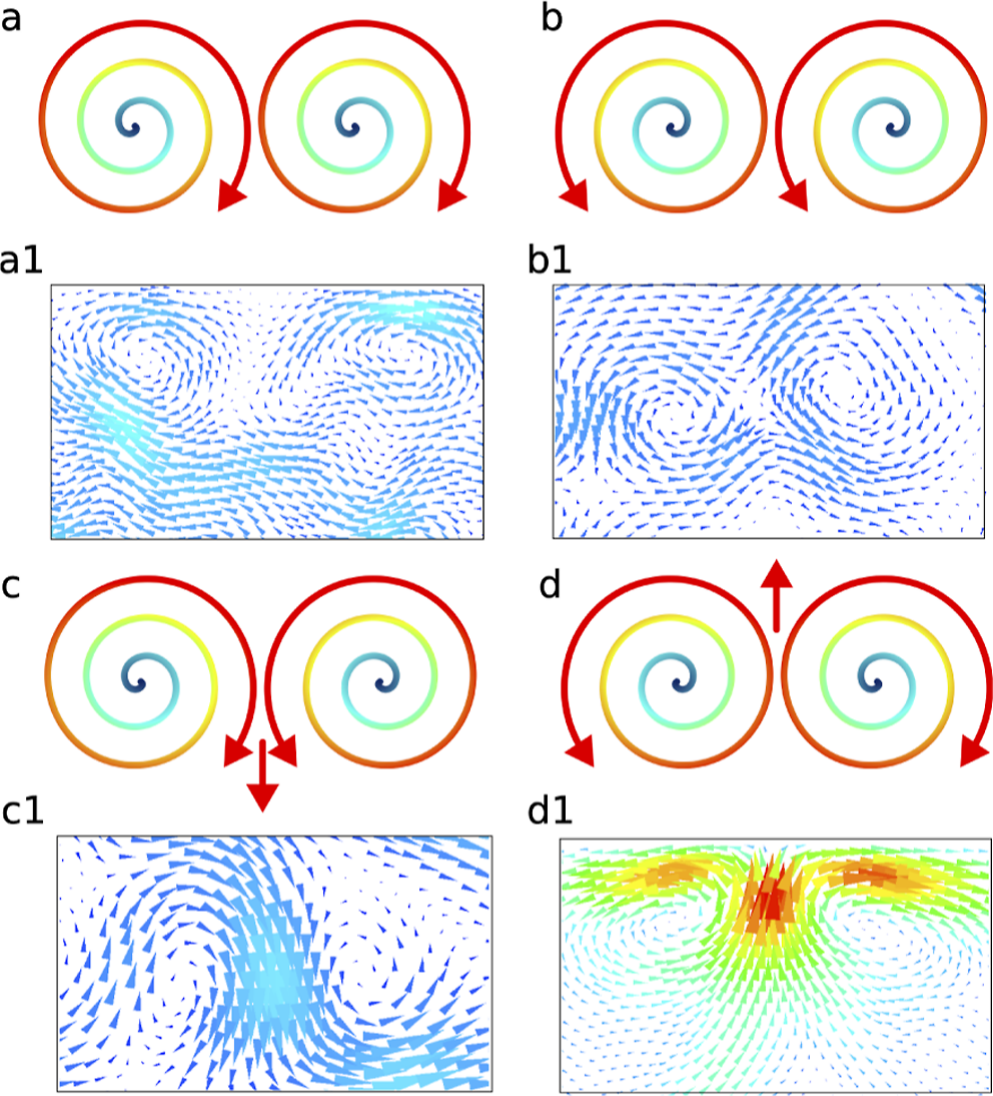
Geometry-dependent pairwise-vortices driven flow. (a) Schematic of a pair of clockwise vortices with zero resultant flow vector. (a1) Results from the simulation with pair unidirectional clockwise vortices without any resultant flow vector. (b) Schematic of pair of anticlockwise vortices with zero resultant flow vector. (b1) Results from the simulation with pair unidirectional anticlockwise vortices without any resultant flow vector. (c) Schematic of the resultant flow vector for a pair of clockwise and anticlockwise vortices. (c1) Simulation following the prediction in (c). (d) Schematic of a strong resultant flow vector for a pair of anticlockwise and clockwise vortices. (d1) Simulation showing the strong resultant flow vector, as depicted in (d).

We found two unique phenomena: self-driven flow and 0FNC state. In Figure 3, some velocity vectors show flow in both directions of water and air without applying any external driving force with a high probability of flow in the direction against the gravity from water to air. Figure 4a.o (*d* = 70 nm, σ = 1.7 μm) represents the systems where the cloud of high-velocity molecules exist and the velocity vectors are pointed toward the air volume fraction. Figure 4b.o (*d* = 70 nm, σ = 2.5 μm) represents the systems with no flow in the air, and the strong back-flow in water creates vertices throughout the boundaries in the water phase. The velocity vectors show several asymmetric swirls and sudden changes in directions with a turbulent nature. In Figure 4c.o (*d* = 50 nm, σ = 2.8 μm), a system with a significant flow difference pattern in the air phase. There are also cases where back-flow toward the water phase is initiated but is relatively less prominent than the velocity vectors toward the water phase. The velocity stream lines in Figure 4d.o (*d* = 70 nm, σ = 1.7 μm) shows highest magnitude of 8.66 × 10^–12^ m/s at the nanopore in the water phase. When the position of nanopore changes to σ = 2.5 μm, in Figure 4e.o (*d* = 70 nm, σ = 2.5 μm), the highest magnitude of velocity streamlines is 4.35 × 10^–12^ m/s. In Figure 4f.o (*d* = 50 nm, σ = 2.8 μm), the velocity streamlines are prominent through the nanopore with a magnitude of 8.66 × 10^–12^ m/s. In Figure 3, the velocity distribution of the multiple nanofluidic pore system becomes more complex than the single nanofluidic pore system. This is indicative that the molecular species in the (*NVT*) ensemble distribute their kinetic energy in a way to maintain the number of evaporated molecules and the number of condensing molecules in equilibrium. The vortex pairs and their vector orientation in these systems are very significant in determining net flow through the nanopores. The disruption of equilibrium at the interface and the backflow through the nanofluidic pore to the water domain may lead to the phenomenon of cavitation^30^ which hinders the further flow which is not uncommon in transport through confined nanofluidic pore systems. Usually, the variations in contact angle and the liquid pressure at the interface control the evaporation process to a great extent and heat is to be supplied for it to sustain.^29^Figure 5 gives the schematic representation of self-driven flow in these system. In Figure 5a,b, there is no net flow due to the cancellation of equal and opposite vector components. Whereas in Figure 5c,d, the resultant position vector is given by
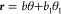
14where *b* and *b*_1_ determines the distance between the spiral loops of each spiral within the vortex pair spirals. Then, eq 13 with the correction term becomes
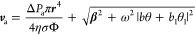
15and for systems with no flow, eq 13 becomes
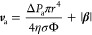
16where **β** is the constant velocity component along the perpendicular direction of nanopore throat. These vortex pairs are evident throughout every simulated system regardless of *d* and σ (as in Figures 5(a1–d1) and S3).

We recognize the potential concern regarding the validity of adopting a continuum approach in a system with a large Knudsen number. The Knudsen number, which characterizes the ratio of molecular mean free path to characteristic length scale, is indeed an important parameter in micro- and nanoscale fluid dynamics. In our study, we have employed a continuum approach as it provides valuable insights into the behavior of nanofluidic flows under the conditions we have considered (convergence of simulations are shown in Figure S5). While it is true that the Knudsen number for our system can be large and slip or rarefied gas effects may become significant in such cases, it is important to note that the continuum approach serves as a valuable first step in understanding the underlying physics. We also recognize that the slip regime and rarefied gas effects are intriguing topics in nanofluidic research, and we plan to explore these aspects in future investigations. The current study lays the foundation for understanding the complex fluid dynamics at the nanoscale, and our findings contribute to the broader understanding of nanofluidic phenomena. The significant reduction in surface tension and surface conductivity in nanopores (compared to that of bulk liquid) is a frequent topic of discussion.^31,32^ The decreasing pore size to nanometre length-scale impacts the hydrogen bonding network in water as a result has lower coordination number of bonding water molecule than the bulk water.^33^ Due to this enhanced affinity of water molecules for the silica surface has a large impact on the system’s molecular dynamics and makes the relationship between the effects of surface tension and nanoconfinement much smaller.^34^ In the modified Laplace–Young equation as in^35^ which considers the solid–liquid interfacial tension, thermal energy exchange, and variation in molecular dynamics in a nanofluidic channel, the pressure gradient of the whole system has significant dependency on the diameter of the transport channel, and the pressure is in the range of megapascal. This changes when phase transition occurs and the nanochannel is connected to a bulk liquid, which has dimensions much greater than that of transport channel as in our case (maximum width of channel = 70 nm, maximum length of channel = 100 nm, and maximum length of bulk reservoir = 6 μm). Magda et al. shows in Lennard-Jones liquid of 2-12 molecules-wide pores that the solvation force on interfacial pores of 9.5 liquid diameter wide pore are equal to the pressure of bulk liquid which is in equilibrium with the pore.^36^ However, this may vary for interfacial tension of pores with smaller width less than 9.5 liquid diameter depending on the pressure.^36^ Similarly, the effect of breakdown of Navier–Stokes formulations and equation of continuity on flow dynamics of microfluidic systems needs to be considered, which is addressed in a following study by us.^37^ However, further study on time-dependent transient states needs to be explored for obtaining greater insights into the exact time-frames and boundary condition to determine the exact point when continuum breaks, which is highly computationally expensive and requires separate attention.

In this study, we consider the mean free path and Knudsen number as prerequisites to the physics of the upcoming studies. We have built a basic structure of a nanoconfined system with critical values to address the physics of molecular nanofluids at the classical–quantum interface. Hence, at this point it may not be appropriate to generalize the results with a critical parameter or nondimensional constant. The main objective of this study is to use computational and theoretical means to not only predict the molecular fluid dynamics but also create a potentially innovative tool of solid-state nanopore fluid dynamics. While our work primarily employs continuum mechanics and analytical methods, it is noteworthy that similar phenomena have been observed using MD simulations in prior research.^4^ Due to the significant computational complexities associated with MD simulations at the scales considered in our study, we opted for a continuum approach. Our results follow the finding of Zou et al.^4^ and extends it beyond the scale and capacity of their study while finding global impact of nanopores in a bulk system. We use the term 0FNC to describe a unique phenomenon observed in our nanoscale fluid dynamics, which exhibits complex and seemingly chaotic behavior despite the absence of classical turbulence. It is important to note that this terminology is used to characterize the specific flow patterns and instabilities observed in our system and does not imply that we are modeling or simulating classical turbulence, as our adopted model does not explicitly account for turbulence effects.

## Conclusions

In short, this study of nanofluidic systems unveils two intriguing phenomena: self-driven flow and the 0FNC state. Self-driven flow, as observed in Figure 3, reveals the unexpected occurrence of fluid motion without the need for external driving forces. Notably, we found a high probability of flow against gravity from water to air, challenging conventional expectations. Figure 4a.o demonstrates the presence of high-velocity molecular clouds, where velocity vectors point toward the air phase. Conversely, Figure 4b.o illustrates situations where the air remains stagnant, resulting in strong backflow within the water phase and creating vortex structures along the boundaries. These velocity vectors exhibit asymmetrical swirls and sudden directional changes, indicative of a turbulent behavior. Figure 4c.o presents a system with significant airflow differences in the air phase. While instances of backflow into the water phase do occur, they are less prominent than the vectors directed toward the water phase. Notably, the highest recorded velocity magnitude of 8.66 × 10^–12^ m/s at the nanopore occurs in Figure 4d.o. The complex nature of the velocity distribution in systems with multiple nanofluidic pores, as shown in Figure 3, underscores the role of the molecular species’ kinetic energy distribution in maintaining equilibrium between evaporated and condensing molecules. The formation and orientation of vortex pairs are pivotal in determining the net flow through these nanopores. The potential for disruption of equilibrium at the interface and back-flow through the nanofluidic pore into the water domain raises intriguing possibilities, such as cavitation,^30^ which can hinder further flow—a phenomenon not uncommon in confined nanofluidic pore systems. Our study sheds light on the intricate interplay between surface tension, conductivity, and confinement in nanofluidic systems. The reduction in surface tension and conductivity at the nanoscale^31,32^ significantly impacts the hydrogen bonding network in water, resulting in a lower coordination number of bonded water molecules compared to bulk water.^33^ This enhanced affinity of water for silica surfaces influences molecular dynamics, with important implications for pressure gradients.^35^ While our research contributes to understanding fluid dynamics at the classical–quantum interface, it is essential to recognize that our findings are preliminary. The complex nature of these systems, encompassing components that defy conventional viscosity, hints at the interplay between anharmonic and harmonic elements within the same system at room temperature.^38^ To conclude, our study explores the nontrivial behavior of Rayleigh–Taylor instability in sub-100 nm fluidic pores, showcasing the potential for self-driven flow and flow control without external forces. These discoveries open doors to fundamental research in nanoscale fluid dynamics and bear relevance to fields such as sustainable energy production, nanorobotics, biomolecular diagnostics, and the broader physics of fluid dynamics.

## Supporting Information Available

List of notations used in the work, governing differential equations, detailed flow velocity of single nanofluidic pore systems, major velocity distribution patterns, convergence information on the simulations, and flow dynamics through the nanopores at the acetone–air interface (PDF)
