# Supplementary material for: Active Solid-State Nanopores: Self-Driven Flows/Chaos at the Liquid–Gas Nanofluidic Interface
Source: Langmuir. 2023 Nov 29;39(51):18889–98. doi: 10.1021/acs.langmuir.3c02776 (PMC10753882; doi:10.1021/acs.langmuir.3c02776)
Supplement: Supplementary file 1 — la3c02776_si_001.pdf [file la3c02776_si_001.pdf]

Active solid-state nanopores: Self-driven flows/chaos at liquid-gas nanofluidic interface

SUPPORTING INFORMATION

Vinitha Johny[1,2] and Siddharth Ghosh[1,2,3,4*]

[1] *International Center for Nanodevices, INCeNSE-TBI, Indian Institute of Science Campus, Bangalore 560 012, Karnataka, India.*

[2] *Open Academic Research Council, Hooghly 712 235, West Bengal, India and Open Academic Research UK CIC, Cambridge CB3 1AT, UK.*

[3] *International Center for Nanodevices, High Tech Campus Eindhoven, 5656 AE Eindhoven, The Netherlands.*

[4] *Department of Applied Mathematics and Theoretical Physics, University of Cambridge, Cambridge CB3 0WA, UK.*

E-mail: sg915@cam.ac.uk

Notations

Table S1: Notations used in the calculations and derivations.

σ	Position of nanopore from the reference edge
d	Diameter of the Nanopore
γ	Phase volume fraction in the multiphase flow
ρ	Phase density
κ	Phase variable
$\boldsymbol{v}$	Phase velocity
M	Mass transport through the nanopore
$\bar{\bar{\tau}}$	Diffusion term/stress tensor
$\dot{m}_{lg}$	Mass transfer rate from phase l to phase g
$\dot{m}_{gl}$	Mass transfer rate from phase g to phase l
ϕ	Velocity potential
A	Amplitude of the wave
k	Wave number
a	Acceleration of the molecules
N	Number of evaporating particles from the nanopore
W	Energy corresponding to work function of evaporation
k_B	Boltzmann constant
Γ	Cross sectional area
P	Pressure
η	Viscosity of liquid
r	Radius of nanofluidic pore
L	Flow length of instability
α_e	Element size in the simulation
N_e	Number of elements in simulation
Φ	Area of nanopore
$\boldsymbol{r}$	Position vector
b	Distance constant
$\boldsymbol{\beta}$	Constant velocity component in a vortex pair
(u, v, w)	Velocity components in x, y, z direction
R_e	Reynolds number
E	Energy
k_{eff}	Effective thermal conductivity
suffix $_{g,l}$	Gas phase and liquid phase, respectively
suffix $_{a,s}$	Analytical and simulation, respectively
suffix $_{in}$	Interface

Governing differential equations

The continuity equation is given by:

$$\frac{\partial \rho}{\partial t} + \frac{\partial \rho \boldsymbol{u}}{\partial x} + \frac{\partial \rho \boldsymbol{v}}{\partial y} + \frac{\partial \rho \boldsymbol{w}}{\partial z} = 0 \tag{S1}$$

where (u, v, w) are the velocity components in (x, y, z) direction respectively, t is the time (usually a large time period is considered for steady state simulation), and ρ is the density. This is a fundamental equation in fluid dynamics and represents the conservation of mass for a fluid. In this equation:

- $\frac{\partial \rho}{\partial t}$ represents the rate of change of density (ρ) with respect to time (t).
- $\frac{\partial \rho \boldsymbol{u}}{\partial x}$ represents the rate of change of the density (ρ) times the velocity component ($\boldsymbol{u}$) in the x-direction (x) with respect to x.
- $\frac{\partial \rho \boldsymbol{v}}{\partial y}$ represents the rate of change of the density (ρ) times the velocity component ($\boldsymbol{v}$) in the y-direction (y) with respect to y.
- $\frac{\partial \rho \boldsymbol{w}}{\partial z}$ represents the rate of change of the density (ρ) times the velocity component ($\boldsymbol{w}$) in the z-direction (z) with respect to z.

The equation states that the total rate of change of density within a fluid element is equal to zero, which means that mass is conserved. In simpler terms, it asserts that the change in density at a given point in space and time is equal to the net flow of mass into or out of that point. This equation is a fundamental part of the Navier-Stokes equations, which describe the behaviour of fluid flow. The momentum equation in equation S1 is given by

x-component

$$\begin{aligned} \frac{\partial (\rho \boldsymbol{u})}{\partial t} + \frac{\partial (\rho \boldsymbol{u}^2)}{\partial x} + \frac{\partial (\rho \boldsymbol{u}\boldsymbol{v})}{\partial y} + \frac{\partial (\rho \boldsymbol{u}\boldsymbol{w})}{\partial z} = \\ -\frac{\partial p}{\partial x} + \frac{1}{Re}\left[\frac{\partial \overline{\overline{\tau}}_{xx}}{\partial x} + \frac{\partial \overline{\overline{\tau}}_{xy}}{\partial y} + \frac{\partial \overline{\overline{\tau}}_{xz}}{\partial z}\right] \end{aligned} \tag{S2}$$

y-component

$$\begin{aligned}\frac{\partial(\rho \boldsymbol{v})}{\partial t}+\frac{\partial(\rho \boldsymbol{u}\boldsymbol{v})}{\partial x}+\frac{\partial(\rho \boldsymbol{v}^2)}{\partial y}+\frac{\partial(\rho \boldsymbol{v}\boldsymbol{w})}{\partial z}=\\ -\frac{\partial p}{\partial y}+\frac{1}{Re}\left[\frac{\partial\overline{\overline{\tau}}_{xy}}{\partial x}+\frac{\partial\overline{\overline{\tau}}_{yy}}{\partial y}+\frac{\partial\overline{\overline{\tau}}_{yz}}{\partial z}\right]\end{aligned} \tag{S3}$$

z-component

$$\begin{aligned}\frac{\partial(\rho \boldsymbol{w})}{\partial t}+\frac{\partial(\rho \boldsymbol{u}\boldsymbol{w})}{\partial x}+\frac{\partial(\rho \boldsymbol{v}\boldsymbol{w})}{\partial y}+\frac{\partial(\rho \boldsymbol{w}^2)}{\partial z}=\\ -\frac{\partial p}{\partial z}+\frac{1}{Re}\left[\frac{\partial\overline{\overline{\tau}}_{xz}}{\partial x}+\frac{\partial\overline{\overline{\tau}}_{yz}}{\partial y}+\frac{\partial\overline{\overline{\tau}}_{zz}}{\partial z}\right]\end{aligned} \tag{S4}$$

The energy equation is given by

$$\frac{\partial(\rho E)}{\partial t}+\nabla.(\boldsymbol{v}(\rho E+P))=\nabla.(k_{eff}\Delta T) \tag{S5}$$

where E represents the internal energy, P is the pressure, and T is the temperature, which are mass-averaged variables according to the volume fraction model. The phase parameter p is included to account for multiphase effects, and k_{eff} denotes the effective thermal conductivity.[1]

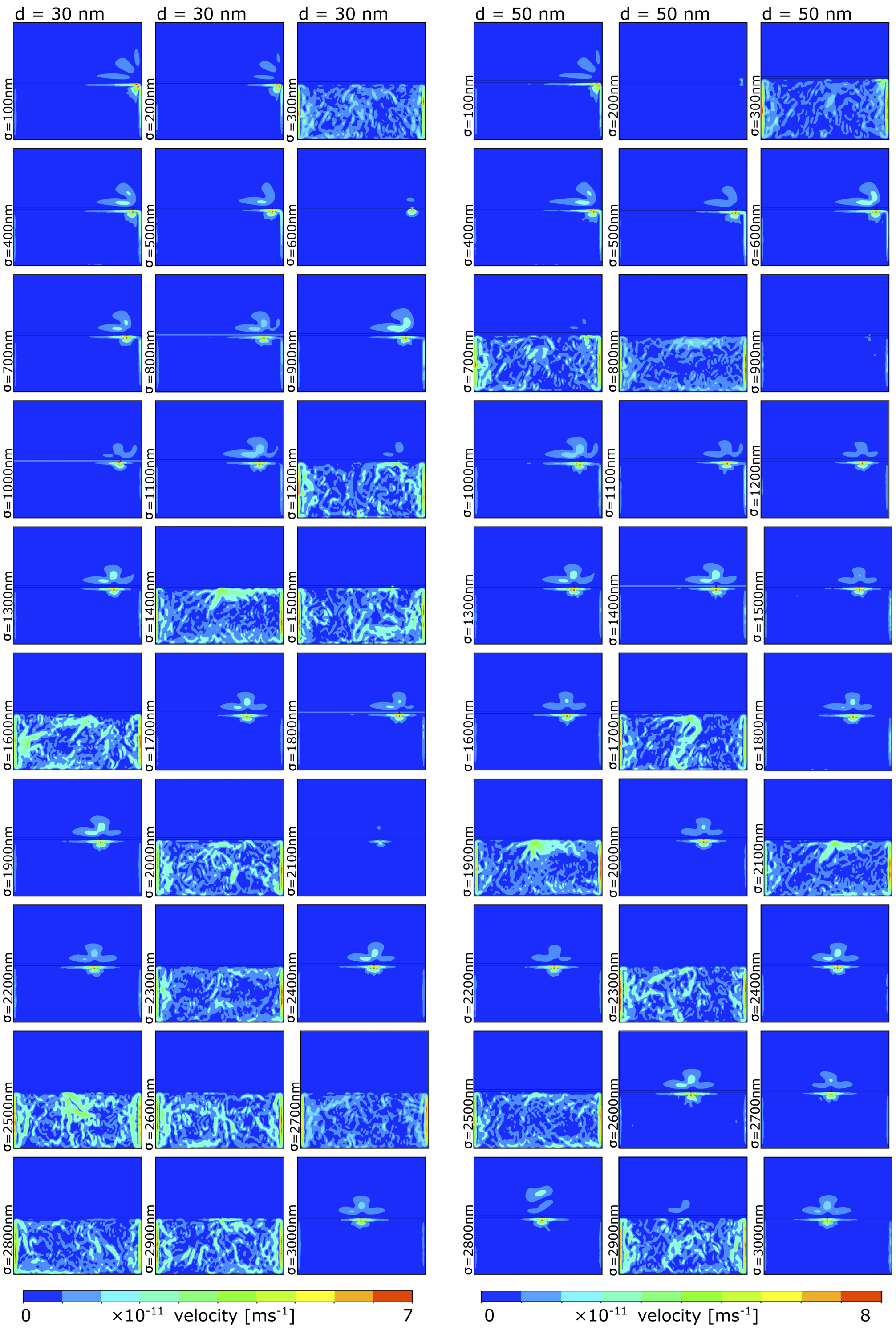

Figure S1: Detailed flow dynamics of 30 nm and 50 nm single nanofluidic pore systems.

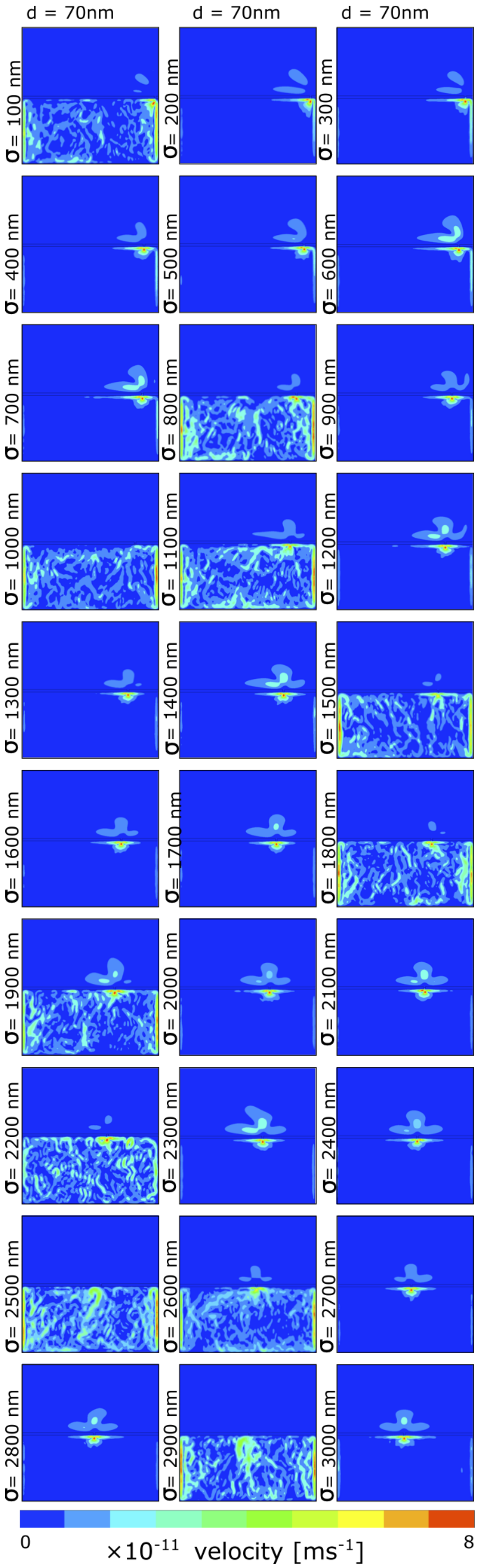

Figure S2: Detailed flow dynamics of 70 nm single nanofluidic pore system.

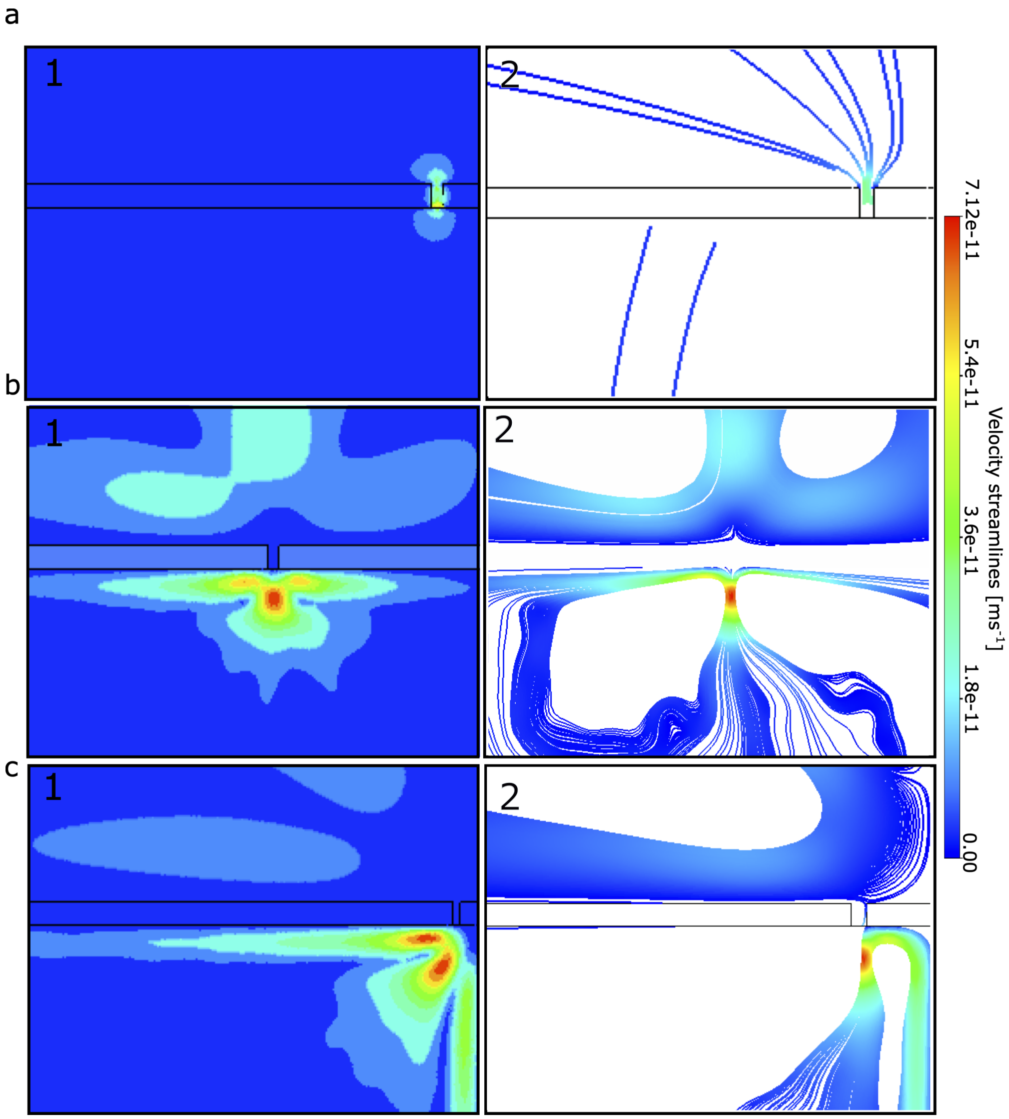

Figure S3: Exemplary cases of local fluidic interactions near the pores (a), (b), (c) Left: Major velocity magnitude distribution patterns and right: velocity streamlines.

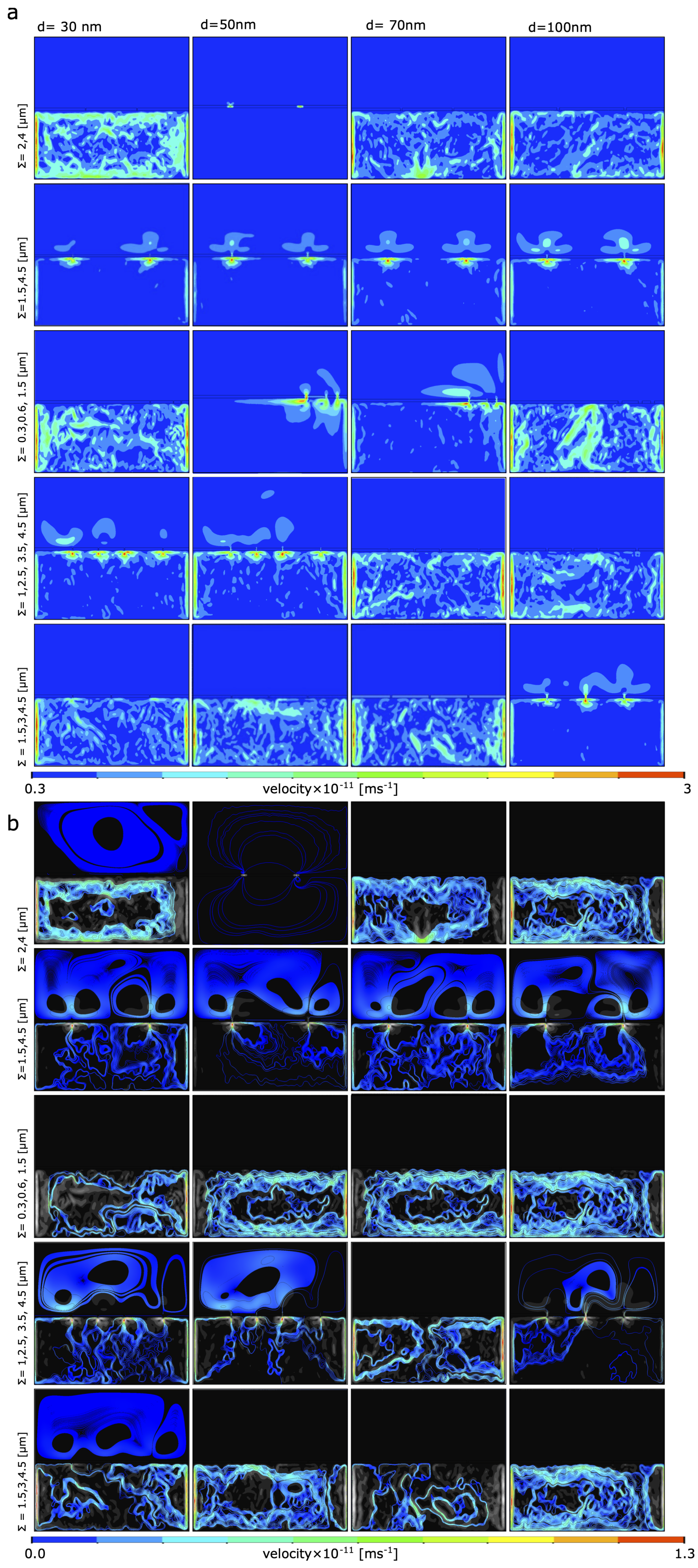

Figure S4: 30 nm, 50 nm, and 70 nm nanofluidic pores' corresponding (a) velocity magnitude distribution patterns and (b) velocity streamline distribution.

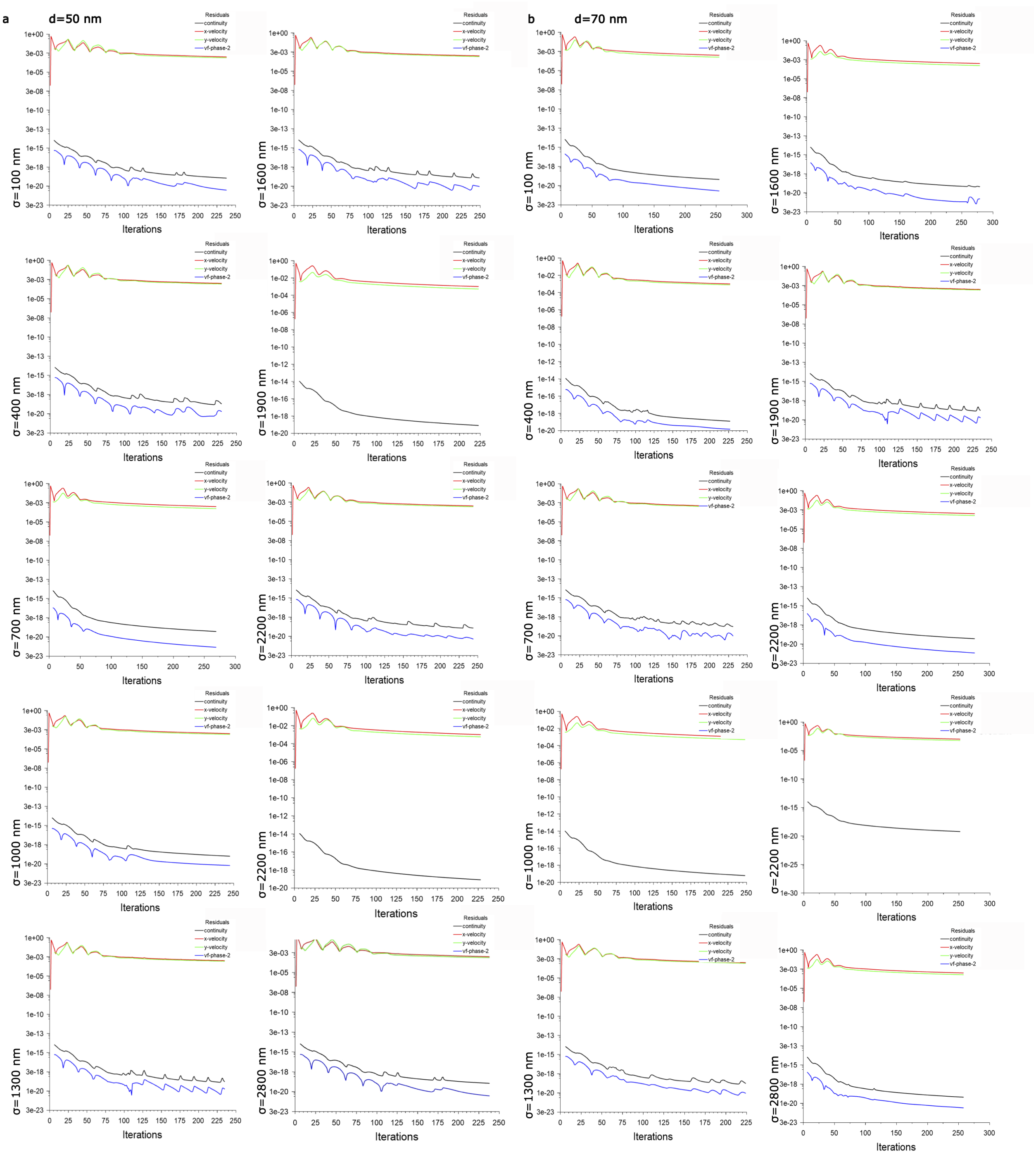

Figure S5: Exemplary convergence data of residuals within the nanopore systems. (a) 50 nm pore size single nanopore systems, and (b) 70 nm pore size single nanopore systems.

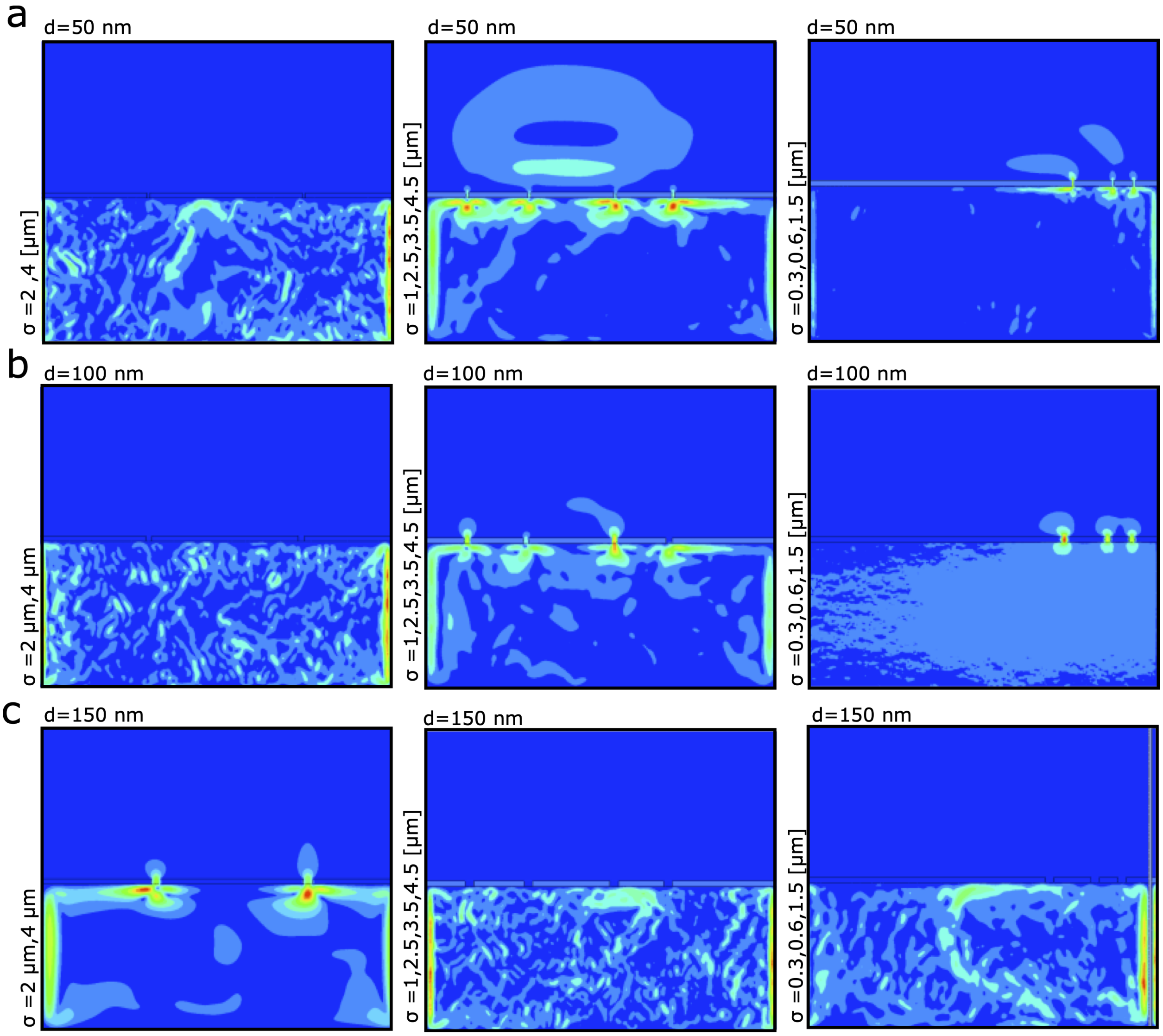

Figure S6: Flow dynamics through the nanopores at acetone-air interface. (a) 50 nm nanopore systems with varying σ, (b) 100 nm nanopore systems with varying σ, (c) 150 nm nanopore systems with varying σ.

References

(1) Manual, U. ANSYS FLUENT 12.0. *Theory Guide* **2009**,
